# Solar photon-Fenton process eliminates free plasmid DNA harboring antimicrobial resistance genes from wastewater

**DOI:** 10.1016/j.jenvman.2021.112204

**Published:** 2021-05-01

**Authors:** Pâmela B. Vilela, Alessandra S. Martins, Maria Clara V.M. Starling, Felipe A.R. de Souza, Giovana F.F. Pires, Ananda P. Aguilar, Maria Eduarda A. Pinto, Tiago A.O. Mendes, Camila C. de Amorim

**Affiliations:** a Research Group on the Environmental Application of Advanced Oxidation Processes (GruPOA), Universidade Federal de Minas Gerais, Engineering School - Sanitary and Environmental Engineering Department, Av. Antônio Carlos 6627, 31270-901, Pampulha, Belo Horizonte, Brazil; b Universidade Federal de Viçosa, Department of Biochemistry and Molecular Biology, Av. Peter Henry Rolfs, Viçosa, Brazil

**Keywords:** *Antimicrobial resistance*, *Plasmids*, *Advanced oxidation processes*, *Neutral photo-Fenton*

## Abstract

This work aimed to assess the elimination and inactivation of resistance-conferring plasmids (RCPs) present in suspension in secondary wastewater by solar photo-Fenton as these are important vectors for the dissemination of antimicrobial resistance. Experiments were performed in synthetic secondary wastewater (SWW) and municipal wastewater treatment plant effluent (MWWTPE). Solar photo-Fenton (50 mg L^−1^ of H_2_O_2_ and 30 mg L^−1^ of Fe^2+^) was carried out for 60 min at neutral pH by applying the intermittent iron addition strategy. The removal of RCPs was assessed by Real-Time Polymerase Chain Reaction (qPCR). The transformation of competent non-resistant *E. coli* was used to evaluate the inactivation of target RCPs harboring antibiotic resistance genes (ARGs) to ampicillin (pSB1A2) or kanamycin (pSB1K3) after treatment and controls. Solar photo-Fenton completely removed RCPs initially present in both matrixes (SWW and MWWTPE), showing enhanced performance compared to the dark Fenton process. Both RCPs were inactivated after 30 min of solar photo-Fenton treatment, while 60 min were necessary to achieve the same effect for the dark Fenton reaction under similar conditions. These results indicate the potential of solar photo-Fenton to improve wastewater quality and reduce the spread of antimicrobial resistance in the environment by hampering the discharge of cell-free RCPs present in suspension in MWWTP onto environmental waters.

## Highlights

• Inactivation of plasmid via solar photo-Fenton assessed via *E coli* transformation.
• Elimination of plasmid from wastewater via solar photo-Fenton assessed via qpCR.
• Solar photo-Fenton removes plasmids carrying antibiotic resistance genes.
• Solar photo-Fenton inactivates plasmids carrying antibiotic resistance genes.
• Higher plasmid removal via solar photo-Fenton in simulated than real wastewater.

## Graphical abstract

**Figure:**
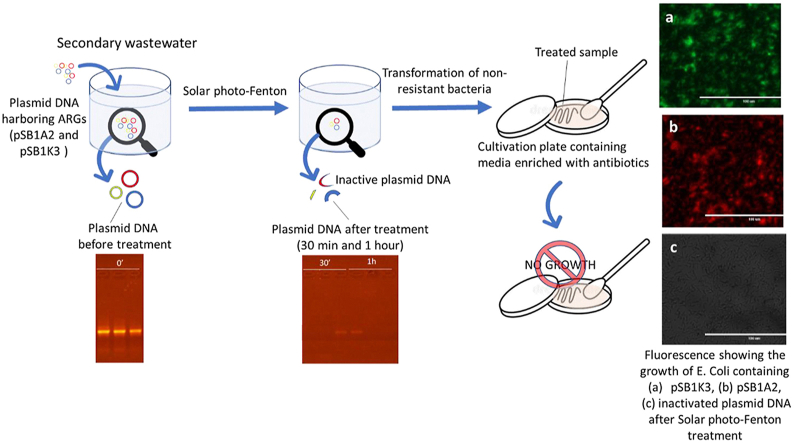

## Abbreviations

AOPs: Advanced Oxidation Processes
AMR: Antimicrobial Resistance
ARB: Antibiotic-Resistant Bacteria
ARGs: Antibiotic-Resistant Genes
CFU: Colony-forming unit
DOC: Dissolved Organic Carbon
GFP: Green Fluorescent Protein
MGEs: Mobile Genetic Elements
MWWTP: Municipal Wastewater Treatment Plant
MWWTPE: Municipal Wastewater Treatment Plant Effluent
NOM: Natural Organic Matter
PCR: Polymerase Chain Reaction
PDS: Peroxydisulfate
PMS: Peroxymonosulfate
qPCR: quantitative Polymerase Chain Reaction
RCP: Resistance-Conferring Plasmids
RFP: Red Fluorescent Protein
SWW: Synthetic Secondary Wastewater

## 1 Introduction

The implications of antimicrobial resistance (AMR) for human and ecological health constitute one of the most critical emerging public health challenges with worldwide proportions. AMR is carried by antimicrobial-resistant bacteria (ARB) and expressed through the activation of antimicrobial resistance genes (ARGs) (Szczepanowski et al., 2009; Vikesland et al., 2019). Despite being a naturally occurring process, AMR may result from acquired mechanisms, referred to as horizontal gene transfer, mainly driven by mobile genetic elements (MGEs) (*i.e.*, plasmids, transposons, and integrons) (Lekunberri et al., 2017; Partridge et al., 2009; Wellington et al., 2013; Xie et al., 2019). Among MGEs, plasmids are naturally occurring circular and extrachromosomal DNA molecules that may carry ARGs and can replicate independently from the host chromosomic DNA. They are transferred horizontally between bacteria or incorporated by bacteria if present as cell-free molecules in the environment (Gokcezade et al., 2014; Rahube et al., 2014; Rosano and Ceccarelli, 2014; San Millan, 2018; C. Zhang et al., 2019a, 2019b). The acquisition of cell-free plasmids harboring ARGs, or resistance-conferring plasmids (RCPs), enables bacteria to evolve rapidly and is one of the critical routes of global dissemination of AMR (Hofer, 2020; Li et al., 2020; Wellington et al., 2013).

Municipal wastewater treatment plants (MWWTP) are among the main hotspots for the development and dissemination of AMR since most of the transformation products and metabolites resulting from the consumption of antibiotics used as human and veterinary medicine are eliminated by excreta. Consequently, these substances are collected in sewer networks and directed to biological reactors present in MWWTP, where they promote a selective pressure that favours ARB and ARGs (Aminov and Mackie, 2007; Baquero et al., 2008; Hiller et al., 2019a; Kümmerer, 2009; Lupo et al., 2012; Rizzo et al., 2013; Wang et al., 2020). As wastewater treatment facilities are usually not designed to remove these contaminants, MWWTP effluent (MWWTPE) still contains a great variety of antibiotics, ARBs, and ARGs. In this matrix ARGs may be either (i) part of bacteria DNA along with other genes, thus being eliminated during disinfection stages, or (ii) present in suspension in MWWTPE as cell-free DNA carried by RCPs and which may persist in the matrix even after the elimination of bacteria (Woegerbauer et al., 2020).

Considering the abundance of ARGs, including those carried by RCPs, in MWWTP effluent (MWWTPE), advanced treatment technologies applied to secondary wastewater are critical stages to promote the elimination and inactivation of ARGs from MWWTPE before proper wastewater disposal (Buckner et al., 2018; Hiller et al., 2019a; Li et al., 2018; Szczepanowski et al., 2009). Although most published studies focus on the removal of ARGs associated with bacterial DNA (Ahmed et al., 2020; Fiorentino et al., 2019; Giannakis et al., 2018; Karaolia et al., 2017; Moreira et al., 2018; Zhang et al., 2016), it is also critical to eliminate cell-free RCPs present in MWWTP, as non-resistant bacteria may acquire them in the soil or river after disposal, thus increasing risks of environmental and human contamination as well as AMR spread (Larsson et al., 2018; Ma et al., 2017; Wellington et al., 2013).

Advanced oxidation processes (AOPs) have been confirmed as effective alternatives for removing ARGs from MWWTPE (Arslan-Alaton et al., 2020; Hiller et al., 2019a; Li et al., 2019; Moreira et al., 2018; C. Zhang et al., 2019a, 2019b). Yet, only a few studies assess the removal of cell-free ARGs carried specifically by RCPs and present in suspension in MWWTPE (Arslan-Alaton et al., 2020; Nihemaiti et al., 2020; Yoon et al., 2018; 2017; M. Zhang et al., 2019a, 2019b) for which concentration may increase after oxidative treatment (Ferro et al., 2016). Besides, extensive studies regarding the acquisition and spread of AMR by RCPs are limited to clinical isolates (Buckner et al., 2018; Hao et al., 2020; Tagliaferri et al., 2020; Wang et al., 2019), and only a few studies have addressed their contribution to AMR in the environment. Yoon et al. (2017) studied the removal and inactivation of cell-free ARGs associated with plasmid DNA (puck4K) via chlorine and UV-C/H_2_O_2_ from the effluent of a conventional activated sludge process. At the same time, Arslan-Alaton et al. (2020) studied the removal of ARB, aphA (kanamycin resistance gene), and tetA (tetracycline resistance gene) located on the plasmid RP4 by UV-C-driven advanced oxidation processes in tertiary treated urban wastewater.

Solar photo-Fenton is a promising alternative for eliminating RCPs from secondary wastewater since it has been proven effective for eliminating some ARGs (Fiorentino et al., 2019; Giannakis et al., 2018; Karaolia et al., 2017). In this process, oxidative radicals, such as hydroxyl radical (HO^●^), is produced during a reaction catalysed by Fe^2+^ ions in the presence of hydrogen peroxide (H_2_O_2_). As Fe^2+^ cycling is enhanced under sunlight, chemical species formed in the system generate an extra route to produce oxidative radicals. One of the main limitations of solar photo-Fenton is related to the pH of operation, as the solubility of Fe^2+^ salts is higher at acidic pH. This may be unravelled by using complexing agents that increase the pH range of iron solubility (*i.e.*, ferrioxalate, EDDS, citric acid, etc.), thus enabling process operation at neutral pH. However, the addition of iron complexes has been associated with an increase in operating costs (Clarizia et al., 2017; Klamerth et al., 2013). As an alternative to using complexing agents, Fe^2+^ may be added intermittently to the system to guarantee an extended availability of this reagent even at neutral pH (Carra et al., 2013; Starling et al., 2021).

In the present study, the potential of solar photo-Fenton (neutral pH, intermittent iron additions) on the elimination and inactivation of RCPs was investigated in synthetic and real MWWTPE. Cell-free RCPs explored in this study carry ARGs that confer resistance to ampicillin and kanamycin. This is unprecedented in the literature as the vast majority of the published studies evaluate the removal of ARGs associated with genomic DNA rather than those carried by RCPs present as cell-free DNA in suspension in MWWTPE (Arslan-Alaton et al., 2020) and do not assess plasmid activity after treatment (Arslan-Alaton et al., 2020; M. Zhang et al., 2019a, 2019b). Also, most published studies targeting RCPs either apply the proposed treatment in water or a synthetic solution (Nihemaiti et al., 2020; Yoon et al., 2018) or to secondary effluent after filtration (Yoon et al., 2017) and asses their removal by other processes (chlorination, UV-C or just UV/H_2_O_2_) rather than solar photo-Fenton (Arslan-Alaton et al., 2020; Nihemaiti et al., 2020; Yoon et al., 2018; 2017; M. Zhang et al., 2019a, 2019b).

## 2 Material and methods

### 2.1 Resistance-conferring plasmids (RCPs)

The plasmids were obtained from 2019 DNA Distribution Kit plates distributed by International Genetically Engineered Machine (iGEM) Foundation (Cambridge, USA) (https://igem.org/Main_Page). Plasmids pSB1A2 and pSB1K3 were used, and the sequence information of each plasmid is found in the iGEM Registry of Standard Biological Parts (http://parts.igem.org) under the accession numbers BBa_J04450, and BBa_I20260, respectively. Plasmid concentration was measured by spectrophotometry at 260 nm and using Qubit Fluorometric Quantification. The vector pSB1A2 is a high copy plasmid containing an ampicillin resistance gene and red fluorescent protein (RFP) gene reporter under a LacZ promoter. Vector pSB1K3 is a high copy plasmid containing a kanamycin resistance gene and green fluorescent protein (GFP) gene reporter under a constitutive promoter.

### 2.2 Synthetic secondary wastewater (SWW)

SWW was used as a model matrix for the assessment of treatment efficiency in order to guarantee the reproducibility of experimental conditions in the different trials as real secondary wastewater may vary according to the sampling campaign. Nutrient solution was prepared by dissolving meat peptone (Kasvi; 160 mg L^−1^), beef extract (Kasvi; 110 mg L^−1^), urea (Synth; CO(NH_2_)_2_; 30 mg L^−1^), NaCl (Sigma-Aldrich; 7 mg L^−1^), CaCl_2_ (Synth; 4 mg L^−1^), MgSO_4_ (Synth; 2 mg L^−1^) and K_2_HPO_4_ (Reagen; 28 mg L^−1^) in ultra-pure water (OECD, 1992). The solution was sterilized after the preparation. Physicochemical characterization of SWW is shown in Table 1. RCPs were added to SWW before tests to reach a final concentration of nearly 10^10^ copies mL^−1^ for total plasmid. This concentration was chosen as it is similar to the total concentration of total cell-free DNA present in real wastewater samples (Yoon et al., 2017).

**Table 1:** Physicochemical characterization of the SWW used as a model matrix (average values; n = 3) to assess the removal of RCPs via solar photo-Fenton.

Parameter	Unit	SWW
COD	mgO_2_ L^−1^	248 ± 10
pH	–	7.5
TOC	mg L^−1^	6.3 ± 4.6
Turbidity	NTU	0.3 ± 0.01
TSS	mg L^−1^	288 ± 111
VSS	mg L^−1^	129 ± 50
TDS	mg L^−1^	158 ± 61
Alkalinity	mgCaCO_3_ L^−1^	26 ± 5
Conductivity	μS cm^−1^	148 ± 0.5

### 2.3 Real municipal wastewater treatment plant effluent (MWWTPE)

MWWTPE was sampled in the output of a secondary settling tank following an activated sludge reactor in an MWWTP located in Brazil, which receives wastewater from 1.5 million inhabitants (290 m^3^ d^−1^), including hospitals, industries, etc. The physicochemical characterization of secondary wastewater is shown in Table 2. MWWTPE samples were sterilized (autoclave) before the spike with RCPs to eliminate all biologically active components. Then, around 10^10^ copies mL^−1^ of total RCPs were added to sterile MWWTPE.

**Table 2:** Physicochemical characterization of the MWWTPE used in solar photo-Fenton experiments performed to remove RCPs.

Parameter	Unit	MWWTPE
COD	mgO_2_ L^−1^	255
pH	–	7.4
TOC	mg L^−1^	12.81
Turbidity	NTU	63
TSS	mg L^−1^	294
VSS	mg L^−1^	132
TDS	mg L^−1^	161
Alkalinity	mgCaCO_3_ L^−1^	57
Conductivity	μS cm^−1^	147

### 2.4 Solar Photo-Fenton treatment

Solar photo-Fenton was performed in a glass reactor (400 mL) placed inside a bench-scale solar simulator chamber (SUNTEST CPS+, ATLAS, 268 W m^−2^) for 60 min (accumulated radiation 5.57 kJ L^−1^). All reactions were conducted using 50 mg L^−1^ of H_2_O_2_ (H_2_O_2_ 29%, Synth) and 30 mg L^−1^ of Fe^2+^ (FeSO_4_ 7H_2_O, Synth). These concentrations of Fe^2+^ and H_2_O_2_ were defined as according to previous studies (Costa et al, 2020, 2021; Starling et al., 2021). Experiments were performed at neutral pH using the intermittent iron addition strategy with Fe^2+^ additions at times zero (15 mg L^−1^), 5’ (5 mg L^−1^), 10’ (5 mg L^−1^), and 15 min (5 mg L^−1^) (Starling et al., 2021). Control systems consisted of solar/Fe, Fe only, solar/H_2_O_2_, H_2_O_2_ alone, and solar irradiation. Samples were withdrawn during reactions to quantify RCP removal (0, 30, and 60 min). Samples were also taken for the quantification of residual hydrogen peroxide using the metavanadate method (Nogueira et al., 2005) and iron (Fe^2+^) (*o*-phenantroline method) (ISO 6332:1988). Catalase enzyme (460 mg L^−1^ in phosphate buffer; Sigma-Aldrich) was added to each sample (0.2 mL of catalase solution for 2 mL of the sample) for residual hydrogen peroxide consumption to stop reactions prior to these analyses. All experiments carried out in SWW and MWWTPE were performed in triplicates.

### 2.5 Extraction of RCPs

Plasmid DNA extraction was performed using the phenol-chloroform–isoamyl alcohol method adapted from Takeuchi et al. (1997). Initially, 300.0 μL of chloroform (CHCl_3_; Anidrol) and 12.5 μL of isoamyl alcohol (C_5_H_12_O; Anidrol) were added to 500 μL of the sample, which was homogenized by inversion and centrifuged at 1200×*g* for 5 min at 4 °C. The upper phase was recovered, and 1:1 solution of absolute ethanol (CH_3_CH_2_OH, Neon) was added, incubated for 10 min at 4 °C, and centrifuged at 12000×*g* for 20 min at 4 °C. The supernatant was then discarded, and 100 μL of ethanol, 70%, was added to the sample, homogenized, and centrifuged at 12,000×*g* for 20 min at 4 °C. Once more, the supernatant was discarded, and the final pellet was placed in the oven to dry at room temperature. Finally, the pellet was resuspended with 20 μL of DNA-free water (H_2_O, Sigma-Aldrich). The resulting concentration of plasmid DNA was determined in a Nanodrop spectrophotometer at 260 nm.

### 2.6 Real-time PCR analysis (qPCR)

The number of copies of RCPs per sample was assessed by real-time PCR using the absolute standard curve method. The VF-2 (5′-TGCCACCTGACGTCTAAGAA-3′) and VE-R (5′-ATTACCGCCTTTGAGTGAGC-3′) primers were used to quantify both plasmids. These primers were designed to recognize specific sequences present in both RCPs which are not associated with the ARGs carried by these plasmids, thus avoiding any interference involving the natural occurrence of these genes in real matrices. The ability of these primers to amplify the two RCPs was previously checked by conventional PCR. A standard curve using a series of RCP dilutions was constructed to calculate the efficiency of each pair of primer to and quantify both RCPs in synthetic and real effluents. qPCR was prepared with 1 μL of genomic DNA template with 10 ng of DNA, 5 μL of SYBR Premix Ex Taq (Promega, EUA), 1 μL of forward and reverse primers, 0.1 μL of dye, and 2.9 μL of DNA-free water (Sigma-Aldrich). Amplification was performed in Light Cycler 480 Real-Time PCR System (Applied Science) using the following program: 95 °C for 10 min, then 40 cycles (95 °C for 30 s and 60 °C for 1 min) followed by a gradual denaturation for the elaboration of the melting curve, with an increment of 1 °C per minute until the temperature reached 95 °C (de Paiva et al., 2019). This experiment was carried out in triplicates.

### 2.7 Cultivation of *E. Coli* competent cells and plasmid transformation

As the oxidation of RCPs may result in cell-free DNA containing intact fragments of the PCR amplification region, inactivated plasmids may continue to amplify in PCR even after damaged. Therefore, the transformation of non-resistant competent *E. coli,* which did not contain the RCPs explored in this study, was used as a method to evaluate the ability of plasmids present in SWW samples after solar photo-Fenton treatment to induce antibiotic resistance. This method was only performed for samples obtained during experiments with SWW as it is laborious and qualitative compared to qPCR.

Briefly, competent bacteria *E. coli* BL21 were prepared using 0.1 M magnesium chloride solution (MgCl_2_–CaCl_2_, Sigma-Aldrich), and competent cells were then transformed with 10 ng of plasmids by the heat-shock method (Chan et al., 2013). The ability of RCP to induce bacteria resistance to antibiotics and produce GFP and RFP reporter color or fluorescence was also evaluated by microscopy and SpectraMax microplate reader as described in Tagliaferri et al. (2020). The experiment was performed in biological triplicates and technical duplicates, and the number of colony-forming unit (CFU) was measured in each experiment.

### 2.8 Statistical analysis

Statistical analyses were performed using GraphPad Prism version 5.0. One-sample Kolmogorov-Smirnoff test was used to evaluate whether the data followed normal distribution. A nonparametric one-way analysis of variance (ANOVA) test was used to compare the means of each experimental value to time zero (non-treated sample) inside the same experimental group with Bonferroni correction for multiple hypotheses. Differences were considered statistically significant at *p <* 0.05.

## 3 Results and discussion

### 3.1 Removal of RCP via Solar Photo-Fenton in SWW

**Fig. 1:**
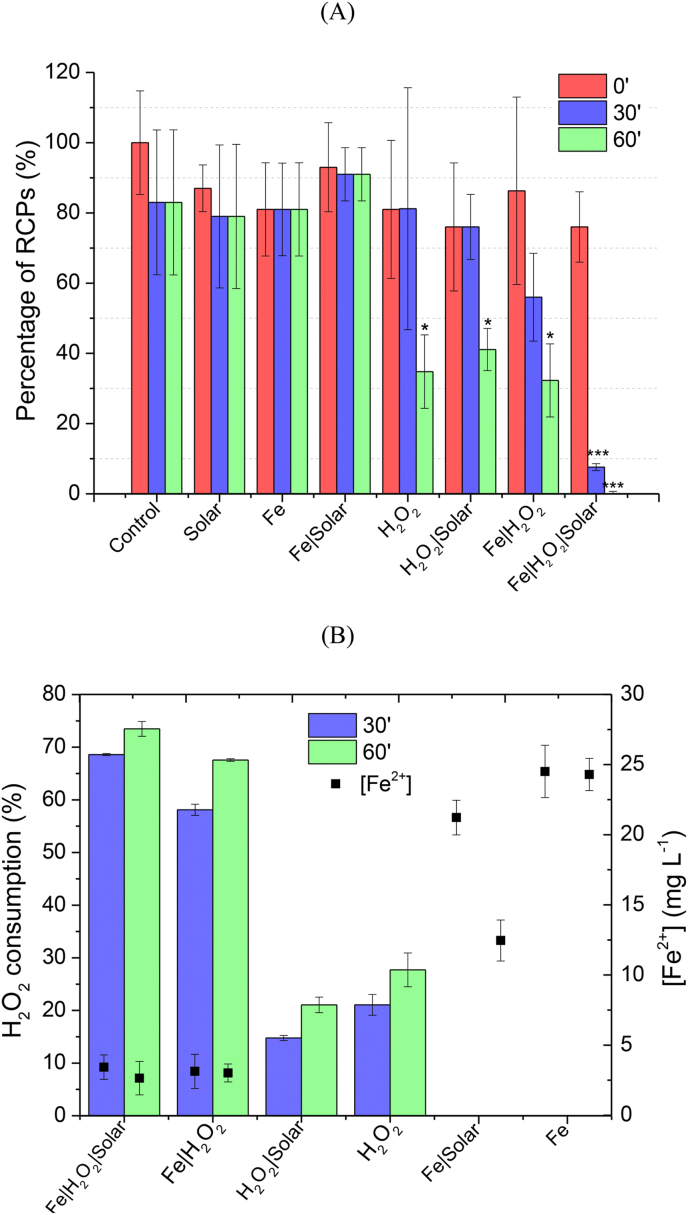
Percentage of RCPs (A), peroxide consumption, iron concentration (B) after 30′ and 60′ of reaction by Fenton, Solar photo-Fenton, and controls carried out in SWW. The symbol * and *** represent significant statistical differences with p < 0.05 and p < 0.001 by nonparametric one-way analysis of variance (ANOVA) test with Bonferroni correction compared to the time 0′ for each experimental group, respectively.

In order to evaluate the efficiency of solar photo-Fenton to remove RCPs harboring antibiotic-resistant genes from wastewater, the qPCR method was used to quantify their presence in samples before and after treatment and controls. Fig. 1A shows the percentage of RCPs present in SWW samples after 30 and 60 min of reaction by Fenton, solar photo-Fenton, and controls. As it may be observed in Fig. 1A, qPCR assays did not detect any copies of RCPs (copies mL^−1^) after 1 h of solar photo-Fenton (accumulated radiation 5.75 kJ L^−1^), thus confirming the efficiency of this process on total elimination of RCPs carrying resistance genes. Similar results were found in previous works which assessed the removal of total ARGs (associated with cell-free plasmid DNA and to bacteria DNA) present in MWWTPE by solar photo-Fenton (Fiorentino et al., 2019; Giannakis et al., 2018; Karaolia et al., 2017; Moreira et al., 2018; Zhang et al., 2016). These studies indicated that the removal of total ARGs by solar photo-Fenton varies according to each ARG, matrix composition, and operational conditions (Fiorentino et al., 2019; Giannakis et al., 2018; Zhang et al., 2016). Also, solar irradiation alone could not remove total DNA (Giannakis et al., 2018), as also observed in this study for the removal of cell-free RCPs (Fig. 1A). Besides, Ferro et al. (2016) observed an increase in cell-free ARGs present in suspension in wastewater samples after UV/H_2_O_2_. Thus, confirming the need for studies targeting cell-free RCPs carrying ARGs to prevent the spread of antimicrobial resistance through wastewater discharge.

Despite the increase observed for cell-free ARGs associated with RCPs present in suspension in MWWTPE samples even after biological treatment, disinfection, and advanced oxidation (Dong et al., 2019; Ferro et al., 2016; Hiller et al., 2019b; Liu et al., 2018), no studies have previously targeted specific removal of cell-free ARGs from MWWTPE via solar photo-Fenton as done in the present study. The effective removal of cell-free RCPs has only been confirmed for treatments under UV-C irradiation in the presence of oxidants (hydrogen peroxide, peroxymonosulfate, or peroxydisulfate) (Arslan-Alaton et al., 2020). UV_254_/H_2_O_2_ and chlorine were studied to remove and inactivate cell-free ARGs associated with plasmid DNA (puck4K) from the effluent of a conventional activated sludge process (Yoon et al., 2017). Arslan-Alaton et al. (2020) demonstrated the high efficiency of oxidant/UV-C treatments, which resulted in complete removal of genomic and plasmid DNA. In Yoon et al. (2017), results indicated that gene structure influences the reduction of ARGs associated with cell-free DNA via chlorination.

Meanwhile, similar removal rates were observed for different ARGs carried by plasmids under UV_254_ or UV_254_/H_2_O_2_ (Yoon et al., 2017). For instance, a 4-log reduction of ARGs required UV fluences between 60 and 130 mJ cm^−2^. Besides, UV-induced ARG damage occurred 1.7-fold faster for the cell-free ARGs when compared to intracellular ARGs in filtered MWWTPE, which showed lower organic matter concentrations than the SWW used in this study. Results obtained in the present work are compatible with results reported in mentioned references previo since the removal of cell-free RCPs was achieved after 30–60 min of treatment. Even so, the use of solar radiation, as proposed in this study, has a range of advantages compared to artificial UV lamps, both from economic and environmental standpoints (Oturan and Aaron, 2014).

Fig. 1 Percentage of RCPs (A), peroxide consumption, iron concentration (B) after 30′ and 60′ of reaction by Fenton, solar photo-Fenton, and controls carried out in SWW. The symbols * and *** represent significant statistical differences with p < 0.05 and p < 0.001 by nonparametric one-way analysis of variance (ANOVA) test with Bonferroni correction compared to the time 0’ for each experimental group, respectively.

Solar irradiation alone, Fe alone, and their combination (Solar + Fe) did not reach any removal of RCPs. This probably occurred because these systems do not generate reactive oxygen species, thus having low reactivity towards RCPs. Solar disinfection (solar only) and solar + Fe processes were also ineffective for eliminating ARGs associated with DNA present in MWWTPE (Giannakis, 2018; Giannakis et al., 2018). However, these were effective for ARB removal due to intra and extracellular ARB damaging processes with loss of protein function. SWW used in the present study did not contain any viable organism as the goal of this study was to analyze the removal of cell-free RCPs present in suspension in MWWTPE rather than ARB. Still, results obtained here and in referred studies indicate that, due to relative stability of DNA, high oxidative potential and extended contact time are usually required for proper elimination of ARGs, thus being more appropriate to use ARGs rather than ARB as indicators to assess the combat of AMR in wastewater samples (Sharma et al., 2016).

**Fig. 2:**
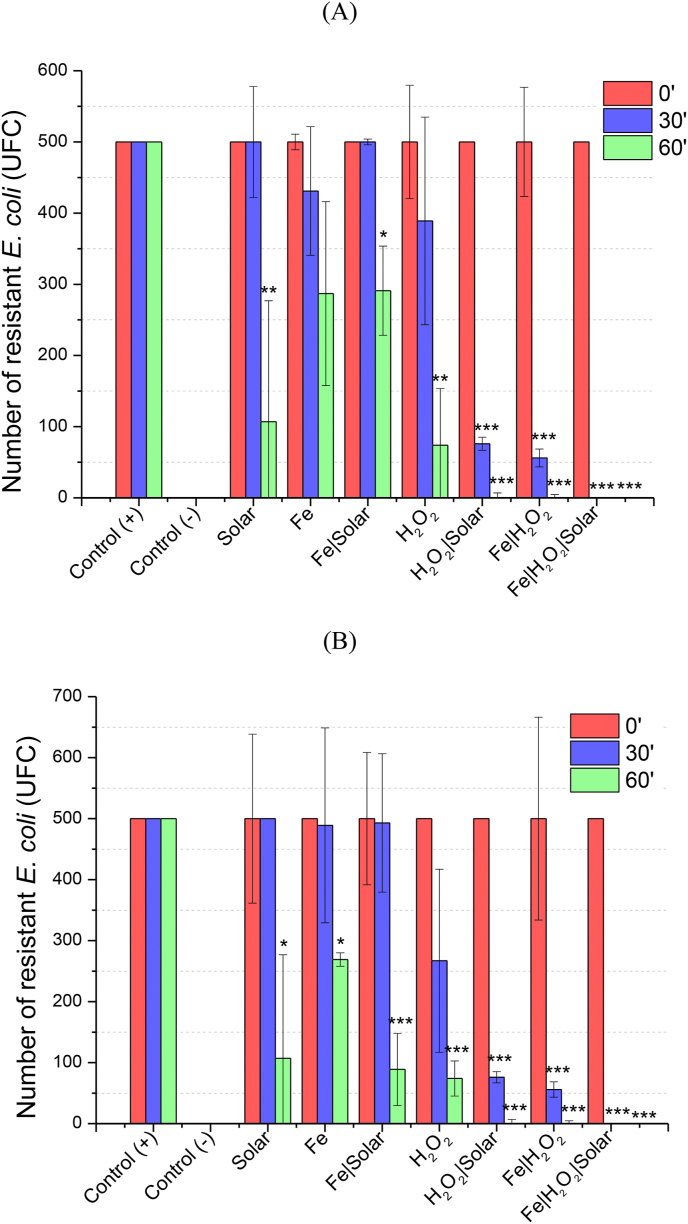
Number of resistant *E. coli* to ampicillin (A) and kanamycin (B) recovery after the transformation with total DNA extracted of each control and experimental treatment sample. The symbol *, ** and *** represent significant statistical differences with p < 0.05, p < 0.01 and p < 0.001 by nonparametric one-way analysis of variance (ANOVA) test with Bonferroni correction in comparison with the time 0′ for each experimental group, respectively.

Regarding the inactivation of RCPs in control experiments performed in SWW, which was detected by the transformation of *E. coli* with DNA extracted from samples after each treatment, solar and solar + Fe did show a significant reduction of functional resistance to ampicillin and kanamycin (Fig. 2 A and B). However, Fe alone was only significantly active towards vector pSB1K3 (kanamycin resistance). These results indicate that, despite not reducing the number of copies of RCPs (Fig. 1A), these controls somehow limit the activity of pSB1K3, which also contributes to the reduction of antimicrobial activity. Although there are no previous studies on the matter to elucidate the possible mechanisms of RCP inactivation by Fe alone, it is suspected that RCPs may have complexed with Fe. This hypothesis relies on the fact that plasmid DNA is negatively charged (Romanowski et al., 1991), and iron is a highly electronegative element. This combination would result in a strong electrostatic attraction between Fe and RCPs (da Silva et al., 2019). Li et al. (2019), thus leading to excellent removal of extracellular ARGs (*e.g*., plasmid DNA) by pre-coagulation integrated with microfiltration as iron enhanced the aggregation of extracellular DNA. Therefore, it was hypothesized that DNA containing negatively charged phosphate groups bind with positively charged Fe hydroxide colloids through electrostatic adsorption and entrapment during the coagulation process.

Fig. 2 Number of resistant *E. coli* to ampicillin (A) and kanamycin (B) recovery after the transformation with total DNA extracted of each control and experimental treatment sample. The symbol *, ** and *** represent significant statistical difference with p < 0.05, p < 0.01 and p < 0.001 by nonparametric one-way analysis of variance (ANOVA) test with Bonferroni correction in comparison with the time 0’ for each experimental group, respectively.

In contrast to results obtained in Yoon et al. (2017), who observed no removal of plasmid DNA in the presence of H_2_O_2_ alone, results shown in Fig. 1A reveal that H_2_O_2_ alone and solar/H_2_O_2_ did lead to a significant reduction of RCPs after 1 h. This outcome is probably associated with the oxidative potential of H_2_O_2_ (E^0^ = 1.8 V) and to the formation of hydroxyl radicals, as approximately 20–30% of oxidant were consumed in both systems (Fig. 1B). Meanwhile, H_2_O_2_ consumption in the dark Fenton (Fe + H_2_O_2_) system was nearly 60% (30 min; accumulated radiation 2.71 KJ.L^−1^) (Fig. 1B), thus contributing to 40% removal of RCPs when compared to 20% for solar + H_2_O_2_, as the reaction between Fe and H_2_O_2_ forms hydroxyl radicals (E^0^ = 2.8 V). In the absence of Fe, H_2_O_2_ consumption probably occurs due to its reaction with organic matter and ions present in SWW (COD of 248 mg L^−1^).

Although H_2_O_2_ alone, solar + H_2_O_2,_ and Fe + H_2_O_2_ have the same efficiency of RCP removal (Fig. 1A), total inactivation of cell-free RCPs could only be observed for solar + H_2_O_2_, Fenton, and photo-Fenton processes (Fig. 2A and B). While solar + H_2_O_2_ and the dark Fenton required 1 h to completely inactivate RCPs (Figure 2A and B), 30 min were sufficient for the photo-Fenton system (Fig. 2A and B). This occurs due to higher H_2_O_2_ consumption in this system (60–70%), thus enhancing the formation of highly reactive species capable of damaging and inactivating cell-free RCPs. Also, as shown in Fig. 1B, H_2_O_2_ consumption was faster in the photo-Fenton process when compared to the dark Fenton, which occurs due to enhanced Fe^2+^ regeneration in the presence of light, leading to increased H_2_O_2_ consumption (Malato et al., 2009).

These results show a correspondence between the removal of cell-free RCPs present in suspension in SWW quantified via qPCR and their inactivation, confirmed by bacterial transformation after solar photo-Fenton. Thus, suggesting that the proposed treatment not only damages the structure of RCPs carrying ARGs yet also eliminates their potential to induce bacterial resistance by complete inactivation. To confirm the possibility of applying this treatment in MWWTP, it is also essential to evaluate the effect of proposed treatment conditions on removing cell-free RCPs in suspension in real MWWTPE.

### 3.2 Removal of RCP via Solar Photo-Fenton in MWWTPE

**Fig. 3:**
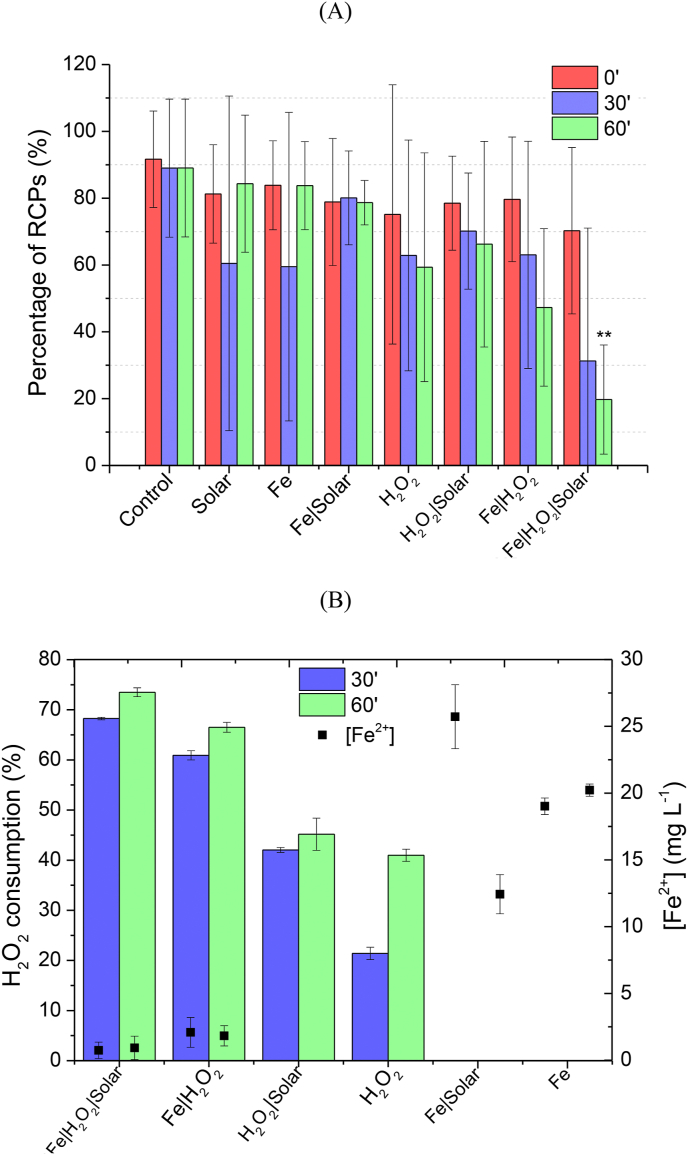
Percentage of RCPs (A), peroxide consumption, iron concentration (B) after 30′ and 60′ of reaction by Fenton, Solar photo-Fenton, and controls carried out in MWWTPE. The symbol ** represents a significant statistical difference with p < 0.01 by nonparametric one-way analysis of variance (ANOVA) test with Bonferroni correction compared with the time 0′ for each experimental group.

Fig. 3A shows the percentage of cell-free RCPs present in samples after 30 and 60 min of reaction by Fenton, solar photo-Fenton, and controls carried out in sterilized MWWTPE. Although real samples are usually more complex and variable, containing several free radical scavenging species (e.g., HCO_3_^−^, Cl^−^, Br^−^, and NO_3_^−^) (Abdelraheem et al., 2020), the SWW used in previous experiments was reasonably consistent to the real MWWTPE applied in this study (Table 1, Table 2). As shown in Fig. 3A, solar photo-Fenton efficiency was limited to ~80% RCP removal in MWWTPE compared to total elimination in SWW. Although both matrixes are remarkably similar concerning natural organic matter content, MWWTPE presented higher turbidity and alkalinity. Therefore, the reduced efficiency obtained in the real matrix is probably due to the light scattering effect promoted by turbidity and the scavenging effect promoted by carbonate ions present in higher concentrations in the real matrix.

In contrast, Nihemaiti et al. (2020) and Yoon et al. (2017) observed similar removal of cell-free DNA via UV-H_2_O_2_ in phosphate buffer compared to real MWWTPE. Yet, the real matrix was filtered before irradiated treatment, thus removing possible effects caused by turbidity. Results obtained in his study for experiments performed in real matrix agree with previous studies, which show that matrix constituents may promote various effects upon reaction kinetics and degradation efficiencies. For instance, Sbardella et al. (2019) reported that removal percentage is reduced by 20–30% due to the scavenging effect of natural components present in secondary effluents under similar treatment conditions. As observed in Fig. 3A, it is evident that photo-Fenton was more effective in removing cell-free RCPs in suspension in real MWWTPE than the dark Fenton process and controls. This occurs as photo-generated ferrous ion participates in the photo-Fenton reaction generating additional HO^●^ radicals, thereby accelerating the oxidation process under irradiation when compared to the dark Fenton process (Kavitha and Palanivelu, 2004; Mosteo et al., 2020), thus resulting in ~80% RCPs removal under irradiation as compared to ~53% achieved by the dark Fenton process.

Fig. 3 Percentage of RCPs (A), peroxide consumption, iron concentration (B) after 30′ and 60′ of reaction by Fenton, Solar photo-Fenton, and controls carried out in RSWW. The symbol ** represents a significant statistical difference with p < 0.01 by nonparametric one-way analysis of variance (ANOVA) test with Bonferroni correction compared with the time 0’ for each experimental group.

Solar irradiation, Fe alone, and Fe + solar, did not reach any removal of RCPs, as these systems do not generate reactive species. Meanwhile, H_2_O_2_ and H_2_O_2_ + solar showed a relatively low percentage of reduction (41% and 34%) compared to results obtained in SWW (67% and 60%), which is possibly a consequence of quenching and light scattering effects occurring in the real sample as previously explained. For the Photo-Fenton process, the removal of cell-free RCPs was nearly 30% lower in MWWTPE than under the same conditions in SWW. This is justified by the HO^●^ scavenging effect promoted by natural components of real MWWTPE, as H_2_O_2_ consumption (Fig. 3B) was similar for both matrixes (~30% in SWW and ~40% in real MWWTPE). Even though the COD of the SWW (248 mg L^−1^) is similar to that observed for MWWTPE (255 mg L^−1^), its composition is more complex as it contains recalcitrant compounds that are more difficult to degrade than those present in the SWW, thus showing higher consumption of oxidative radicals which decrease the availability of these species for the oxidation of target cell-free RCPs present in solution in MWWTPE.

## 4 Conclusions

This study reveals the effectiveness of solar photo-Fenton at neutral pH to remove and inactivate cell-free RCPs present in suspension synthetic and real MWWTPE. As these RCPs carry ARGs that non-resistant bacteria may acquire in the environment, results presented here indicate the potential of solar photo-Fenton to combat the spread of AMR related to secondary wastewater disposal. Solar photo-Fenton promoted total elimination and inactivation of RCPs within 30 min of reaction in SWW. Meanwhile, 60 min were necessary for similar effects in the absence of light. Although Solar + H_2_O_2_ and H_2_O_2_ alone showed effective removal of RCPs (>60%) in SWW, these processes were not able to inactivate RCPs. The role of Fe^2+^ alone on the inactivation of RCP from secondary wastewater must be further elucidated in future studies.

Experiments performed in MWWTPE confirmed the efficiency of the solar photo-Fenton process on the removal (≈80%) of cell-free RCPs present in solution in MWWTPE under the same conditions tested in SWW. Results also indicated the influence of matrix composition upon oxidant and hydroxyl radical consumption as only solar photo-Fenton led to significant (>60%) plasmid removal in real samples.

## Funding sources

This work was supported by the 10.13039/100000865Bill & Melinda Gates Foundation, Seattle, WA [Grand Challenges Exploitations Brazil, grant number 443776/2018–0]; the Foundation of Support and Research of the state of Minas Gerais (10.13039/501100004901FAPEMIG); Coordination of Superior Level Staff Improvement (10.13039/501100002322CAPES); and the 10.13039/501100003593National Council for Scientific and Technological Development (CNPq).

## Role of funding sources

The funding sources were not involved in study design, nor the collection, analysis, and interpretation of data, nor in the writing of the report, nor in the decision to submit the article for publication.

## Credit author statement

Pâmela B. Vilela, Conceptualization, Methodology, Validation, conduction of experimental tests, Formal analysis, Investigation, Writing – original draft and reviewing revised version, Visualization. Alessandra S. Martins, Felipe A.R. de Souza, Giovanna F.F.Pires, Ananda P.Aguilar, Maria Eduarda A. Pinto, Conceptualization, Methodology, conduction of experimental tests, Formal analysis. Maria Clara V. Starling, Tiago A.O.Mendes and Camila C.de Amorim, Conceptualization, Methodology, Validation, Resources, Formal analysis, Investigation, Writing – original draft and writing-reviewed versions & editing, Supervision, Project administration Funding acquisition.

## Declaration of competing interest

The authors declare that they have no known competing financial interests or personal relationships that could have appeared to influence the work reported in this paper.
